# Supplementary material for: Saccharomyces boulardii (CNCM I-745) improves intestinal damage in sepsis by remodeling intestinal flora structure
Source: Front Cell Infect Microbiol. 2025 Jul 31;15:1602792. doi: 10.3389/fcimb.2025.1602792 (PMC12350329; doi:10.3389/fcimb.2025.1602792)
Supplement: Supplementary file 1 [file DataSheet1.zip › summary/summary/8_Advanced_analyse/1_Correlation_analysis/network_0.8.pdf]

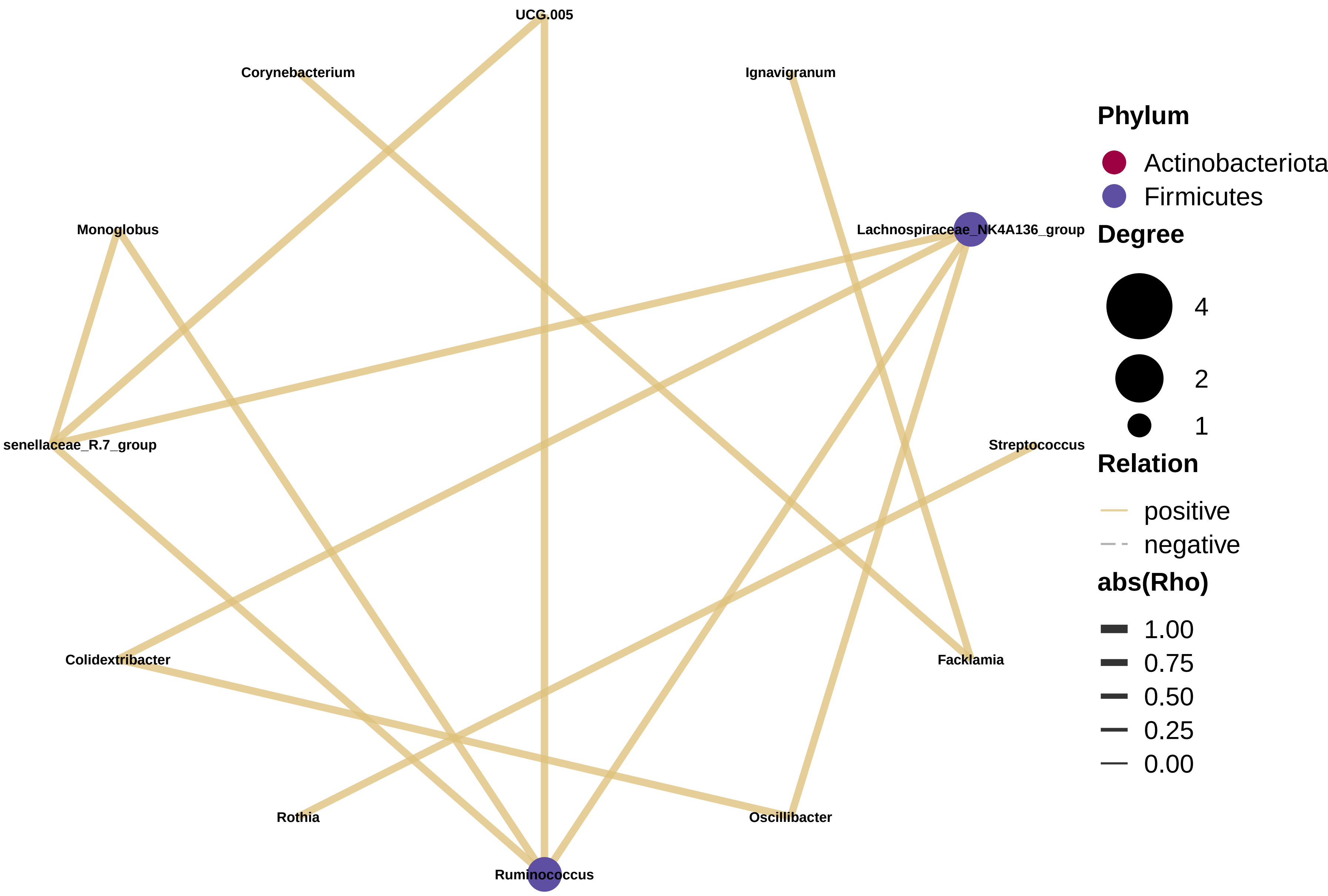
UCG.005
Corynebacterium
Ignavigranum
Monoglobus
Lachnospiraceae_NK4A136_group
senellaceae_R.7_group
Streptococcus
Colidextribacter
Facklamia
Rothia
Oscillibacter
Ruminococcus
Phylum
Actinobacteriota
Firmicutes
Degree
4
2
1
Relation
positive
negative
abs(Rho)
1.00
0.75
0.50
0.25
0.00
